# Circulating Tumour Cell Release after Cement Augmentation of Vertebral Metastases

**DOI:** 10.1038/s41598-017-07649-z

**Published:** 2017-08-03

**Authors:** Malte Mohme, Sabine Riethdorf, Marc Dreimann, Stefan Werner, Cecile L. Maire, Simon A. Joosse, Frederic Bludau, Volkmar Mueller, Rui P. L. Neves, Nikolas H. Stoecklein, Katrin Lamszus, Manfred Westphal, Klaus Pantel, Harriet Wikman, Sven O. Eicker

**Affiliations:** 1 Department of Neurosurgery, University Medical Centre Hamburg-Eppendorf, Hamburg, Germany; 2 Department of Tumour Biology, University Medical Centre Hamburg-Eppendorf, Hamburg, Germany; 3 Department of Trauma-, Hand- and Reconstructive Surgery, University Medical Centre Hamburg-Eppendorf, Hamburg, Germany; 4 Department for Trauma Surgery, University Medical Centre Mannheim, University of Heidelberg, Heidelberg, Germany; 5 Department of Gynecology, University Medical Centre Hamburg-Eppendorf, Hamburg, Hamburg Germany; 6 Department of General, Visceral and Pediatric Surgery, University Hospital and Medical Faculty of the Heinrich-Heine University Dusseldorf, Dusseldorf, Germany

## Abstract

Cement augmentation via percutaneous vertebroplasty or kyphoplasty for treatment of spinal metastasis is a well-established treatment option. We assessed whether elevated intrametastatic pressure during cement augmentation results in an increased dissemination of tumour cells into the vascular circulation. We prospectively collected blood from patients with osteolytic spinal column metastases and analysed the prevalence of circulating tumour cells (CTCs) at three time-points: preoperatively, 20 minutes after cement augmentation, and 3–5 days postoperatively. Enrolling 21 patients, including 13 breast- (61.9%), 5 lung- (23.8%), and one (4.8%) colorectal-, renal-, and prostate-carcinoma patient each, we demonstrate a significant 1.8-fold increase of EpCAM+/K+ CTCs in samples taken 20 minutes post-cement augmentation (*P* < 0.0001). Despite increased mechanical CTC dissemination due to cement augmentation, follow-up blood draws demonstrated that no long-term increase of CTCs was present. Array-CGH analysis revealed a specific profile of the CTC collected 20 minutes after cement augmentation. This is the first study to report that peripheral CTCs are temporarily increased due to vertebral cement augmentation procedures. Our findings provide a rationale for the development of new prophylactic strategies to reduce the increased release of CTC after cement augmentation of osteolytic spinal metastases.

## Introduction

Treatment of metastases represents one of the biggest challenges in oncology^1^. The bone is one of the most frequent sites for metastasis of malignant epithelial tumours^2^. Osseous metastases indicate an advanced metastatic disease stage, which is associated with a high mortality rate and a median survival of less than six months in breast- and prostate cancer patients^2–5^. However, recent advances in oncological treatment options have paved the way for an increased survival in many cancer entities, resulting in a steady increase of long-term cancer survivors^6^. Unfortunately, late metastatic seeding can still be observed after primary therapy. In addition to the overall poor disease outcome, metastases, especially to the bone, are associated with a variety of comorbidities, such as excruciating pain due to pathological fractures, hypercalcemia and symptoms of nerve compression, which severely impede the quality of life of cancer patients^2, 7^. A wide array of symptomatic treatments, including surgical stabilisation procedures or bone-specific drugs such as bisphosphonates and monoclonal-antibodies against RANKL (Denosumab) can slow the disease progression^8, 9^, thereby substantially enhancing the quality of life. With an increasing amount of cancer patients surviving in the metastatic disease stage^6^, surgical treatment options, such as cement augmentation of spinal metastases^10–12^, gain increasing importance and have to be evaluated in the context of systemic tumour cell dissemination.

The spinal column is the most frequent site of bone metastasis in the body^13^. Primarily intended for the apparent and rapid pain relief, cement augmentation due to percutaneous vertebro- (VP) or kyphoplasty (KP) for treatment of spinal metastasis is a well-established treatment with a less invasive nature compared to open spinal surgery^10–12, 14^. However, there is a well-known potential risk of leakage of the liquid cement out of the vertebral body into the surrounding vessels with subsequent embolisation^15–17^. Furthermore, reports about tumour extravasation after VP are known^18^. Presumably, these tumour extravasations spread by one of the above-mentioned routes. Based on these facts we assessed whether elevated intrametastatic pressure during cement augmentation results in increased release of tumour cells into the vascular circulation.

In numerous studies both the number and the persistence of circulating tumour cells (CTCs) are associated with a worse prognosis^19, 20^. Especially in breast cancer the presence of CTCs has been shown to be a strong and independent prognostic factor for both, early stage and metastatic patients^20, 21^.The prognostic and biological role of CTCs detected directly after a biopsy or surgical procedure is, however, still unknown and controversially discussed^22^. The aim of this study was to investigate if cement augmentation of spinal metastases can result in increased release of CTCs and thereby representing a potential risk factor for additional metastatic tumour cell seeding.

## Results

### Patient cohort and tumour characteristics

**Figure 1:**
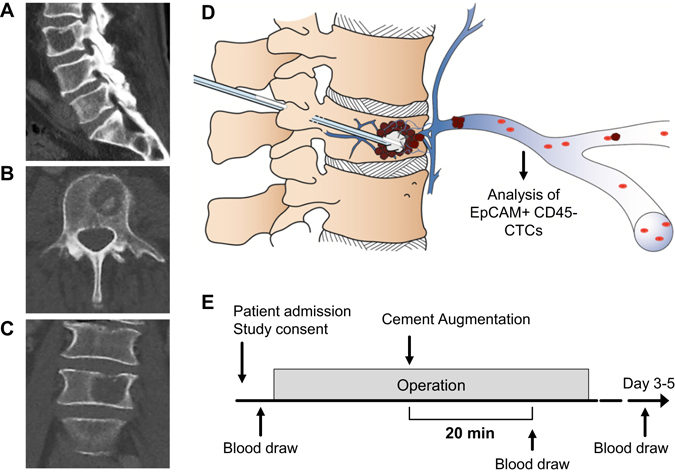
Study evaluating circulating tumour cells (CTCs) in cement augmentation via vertebro- (VP) and kyphoplasty (KP). (**A**–**D**) Radiographic X-ray image in sagittal (**A**), axial (**B**) and coronal (**C**) plane visualizing an exemplary osteolytic vertebral metastases in lumbar vertebra L3. (**D**) Schematic explanation of cement augmentation due to vertebroplasty with subsequent mechanical dissemination of CTCs. (**E**) Illustration of blood draw time-points and study design. Copyright by Sabine Wuttke, UKE, Hamburg.

Our study assessed CTC counts in twenty-one patients (15 female, 6 male) who underwent percutaneous VP or KP procedures for metastatic spinal osteolysis (Fig. 1A–D). Blood was drawn preoperatively, 20 minutes after cement augmentation, and on day 3–5 prior to discharge (Fig. 1E). The mean age was 62.6 years (SD: 11.8 years) with a range from 45 to 83 years. Histopathological diagnosis of the primary tumours confirmed breast carcinoma in 13 (61.9%) patients, in 5 (23.8%) non-small cell lung cancer and one patients (4.8%) each for colorectal-, urothelial- and prostate-carcinoma. In 52.4% of patients vertebral metastases were treated in a setting where only one organ system was affected. Metastases in the spine occurred after 124.9 months (mean, SD: 95.0 months) for breast cancer patients and 6.75 months (mean, SD: 18.3 months) for other histologies after initial diagnosis of the primary tumour (*P* = 0.0017). Patient’s demographics are shown in Supplemental Tables 1 and 2.

Cement augmentation was performed in a total of 34 vertebral bodies, of which 52.9% (*N* = 18) were located in the thoracic-, 44.1% (*N* = 15) in the lumbar- and 2.9% (*N* = 1) in the sacral spine (Tables 1 and 2). In 38.1% (*N* = 8) of patients multiple levels were augmented. Mean metastasis volume was 5.12 ml (SD: 3.51 ml) and the mean injected volume of bone cement was 5.21 ml (SD: 3.68 ml) (Tables 1 and 2).

**Table 1:** CTCs in Patients with Breast-Ca Metastases.

No.	Primary Tumor	Affected level(s)	Proc.*	Cement (total) [ml]	CTC pre OP [/7.5 ml]	CTC 20 min [/7.5 ml]	∆CTC 20 min - preOP	CTC day 3–5 [/7.5 ml]	CTC late FUP [/7.5 ml]
1	Breast-Ca	1x Th	VP	4	6	77	71	0	n.a.
2	Breast-Ca	1x Th	VP	3	16	8	−8	46	3
3	Breast-Ca	1x L	Kypho	3	19	42	23	14	n.a.
4	Breast-Ca	3x Th	VP	4	196	194	−2	80	n.a.
5	Breast-Ca	1x L	Kypho	3.5	1	92	91	0	0
6	Breast-Ca	2x Th	VP	2	0	9	9	0	n.a.
7	Breast-Ca	2x Th	Kypho	4.5	82	139	57	60	1
8	Breast-Ca	1x L	VP	6	0	111	111	0	0
9	Breast-Ca	1x Th, 2x L	Kypho	11	25	151	126	54	n.a.
10	Breast-Ca	2x Th	ioRT Kypho	13	25	35	10	39	n.a.
11	Breast-Ca	1x L	ioRT Kypho	9	0	25	25	1	n.a.
12	Breast-Ca	1x L	VP	4	0	4	4	0	n.a.
13	Breast-Ca	1x Th, 1x L	Kypho	8	20	42	22	7	n.a.

*VP = Vertebroplasty; Kypho = Kyphoplasty; ioRT Kypho = intraop radiation Kyphoplasty. Circulating tumour cell (CTC) counts of breast-cancer group according to procedure and level. L: lumbar, Th: thoracic.

**Table 2:** CTCs in Patients with Carcinoma Metastases.

No. (amount)	Primary Tumor	Affected level	Proc.*	Cement (total) [ml]	CTC pre OP [/7.5 ml]	CTC 20 min [/7.5 ml]	∆CTC 20 min - preOP	CTC day 3–5 [/7.5 ml]	CTC late FUP [/7.5 ml]
14	NSCLC	1x Th	VP	2.4	2	2	0	2	n.a.
15	NSCLC	1x Th, 2x L	Kypho	12	18	62	44	26	n.a.
16	NSCLC	1x Th, 3x L	Kypho	12	10	60	50	10	0
17	NSCLC	1x L	Kypho	8.5	0	4	4	0	n.a.
18	NSCLC	1x Th	VP	3	3	4	1	2	n.a.
19	Colorectal-Ca	1x Th	VP	3	24	42	18	11	n.a.
20	Urothel-Ca	1x S	VP	4	8	11	3	12	n.a.
21	Prostate-Ca	1x L	VP	4	23	40	17	5	n.a.

*VP = Vertebroplasty; Kypho = Kyphoplasty. Circulating tumour cell (CTC) counts of non-breast-cancer group according to procedure and level. L: lumbar, Th: thoracic, S: sacral.

### Release of CTCs due to cement augmentation

**Figure 2:**
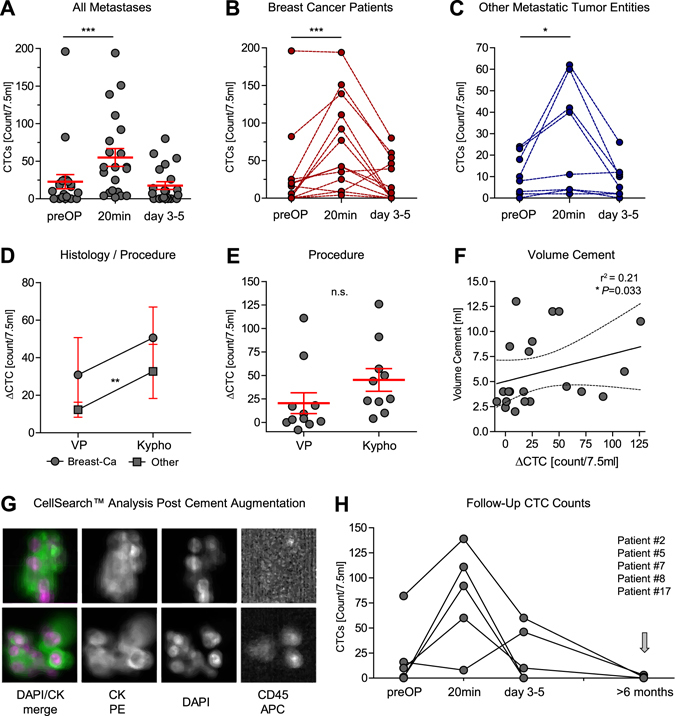
(**A**–**C**) Circulating tumour cell (CTC) counts in 7.5 ml of blood before (preOP), 20 minutes after and on day 3–5 after cement augmentation. *P*-values were determined by generalized linear mixed (GLM)-effect model for repeated measures. Bars and error depict mean CTC count and SE. Subgroup analysis shows connected CTC count course for individual patients, separated for breast-cancer (**B**) and other metastatic tumour entities (C) which are detectable by CellSearch™. (**D**) Increase of CTC counts (CTC_preOP_ − CTC_20min_ = ∆CTC/7.5 ml) compared between kypho- (Kypho) and vertebroplasty (VP) according to histology. (**E**) CTC increase (∆CTC/7.5 ml) by procedure (Vertebro- (VP) vs kyphoplasty (Kypho), bars show mean and SE). Statistical values determined by a GLM-effect model for repeated measures. (**F**) Correlative comparison of CTC increase (∆CTC) with applied cement volume [ml] with linear regression curve fit and 95% CI analysed by Spearman correlation. (**G**) Representative CellSearch™ analysis two showing CTC clusters of two patients and individual K-PE, DAPI, CD45, as well as merged DAPI/K pictures. (**H**) CTC counts course of five patients with long-term follow-up blood draws (>6 months).

CTC analyses demonstrated a 1.8-fold mean increase in CTC count between the preoperative and 20 minutes time-points (standard error (SE): 0.40, preOP vs 20 min *P* < 0.0001) (Fig. 2A). However, this effect had dissipated again after 3–5 days where the level of CTCs was comparable to before cement augmentation (preOP vs day 3–5 *P* = 0.18). The CTC release could be observed in both, breast-cancer (preOP vs 20 min *P* = 0.0009) (Fig. 2B) and non-breast cancer patients (preOP vs 20 min *P* = 0.02) (Fig. 2C). Although the breast cancer patients had 1.4x more CTCs than patients with other tumour entities overall (SE: 0.59, *P* = 0.018) (Supplemental Fig. 1A), the non-breast cancer patients had a CTC increase of 2.0-fold after kyphoplasty procedure (SE: 0.76, *P* = 0.0099) (Fig. 2D). In total, 85.7% (*N* = 18/21) of patients showed an increase of CTC counts due to the surgical procedure.

Patients in which only one organ system, i.e. the bone, was affected by metastases a similar CTC count was found after cement augmentation compared to patients with multiple metastatic sites at the time of surgery (∆CTC median 21 vs 28, *P* = 0.3908, Wilcoxon rank sum test) (Supplemental Fig. 1B). Cement augmentation of lumbar vertebral metastases resulted in a slightly increased CTC dissemination when compared to thoracic metastases (∆CTC median 20 vs 9, *P* = 0.1908, Wilcoxon rank sum test) (Supplemental Fig. 1C), which might have been due to the size/volume of the treated metastases which are in general larger in lumbar vertebral bodies (Fig. 2F and Supplemental Fig. 1). Although only in four patients, we observed CTC clusters (≥2 CTCs) at the 20 minutes time-point, indicative of mechanical forces involved in the CTC release (Fig. 2G).The number of detected clusters and CTCs within the clusters varied between 8 duplets in one patients to 2–12 clusters with 4–12 cells/cluster in the other patients.

### Risk factors for CTC release

During kyphoplasty procedures intratumoural balloon pressure was raised up to 150–200 mmHg in order to stabilize the fractured vertebrae and pre-form a cave for sufficient cement volume. We hypothesised that this might lead to an even higher increased tumour cell release. We did not find an increase in CTC release in patients undergoing cement augmentation after kyphoplasty compared to vertebroplasty alone in the overall patient cohort (*P* = 0.77) (Fig. 2E). Nevertheless, a significant interaction between the non-breast cancer patients and the kyphoplasty procedure was detected, indicating that the release of tumour cells in these patients was indeed associated with the procedure in this subgroup (*P* = 0.0099) (Fig. 2E). The difference of CTC between the preoperative time-point and 20 minutes post-cement augmentation (∆CTC) showed significant, but weak correlation with size of the augmented metastasis (Supplemental Fig. 1D, *P* = 0.0352, Spearman correlation r^2^ = 0.2355). This was also confirmed when correlating cement volume to the ∆CTC (Fig. 2F, *P* = 0.0327, Spearman correlation r^2^ = 0.21).

### Follow-up and CTC analyses

In five patients, follow-up blood samples were drawn for CTC analyses. On average, samples were taken 12.4 months (min 6.9, max 17.8 months, SD: 4.6) after spinal surgery. Although most patients had an immediate increase after cement augmentation procedure, only two out of five patients demonstrated residual CTC positivity during follow-up analyses (Fig. 2H, Tables 1 and 2). Our patient cohorts were characterised by a significant difference in the time of metastatic involvement of the spinal column. Spinal metastases occurred significantly earlier in the non-breast cancer patient group compared to the breast cancer group (mean 6.9 vs. 123.0 months, *P* = 0.0035). In addition, only one case within the breast cancer group demonstrated metastases at initial diagnosis (M1) compared to the majority within the non-breast cancer group, again reflecting the diverse biology of the different cancer entities (Supplemental Tables 1 and 2). However, no difference in metastasis pattern or number of involved levels was observed between the groups. Out of 18 patients with sufficient follow-up (>100 days, mean 332 days follow-up) from the time of surgery, 72.2% (*N* = 13/18) developed new metastases after the cement augmentation procedure (Supplemental Fig. 1E). Most of them were located in the liver (53.8%, *N* = 7/13). Overall 27.8% (*N* = 5/18) died due to progressing disease during follow-up (Supplemental Tables 1 and 2).

### Phenotyping of mechanically released CTCs

**Figure 3:**
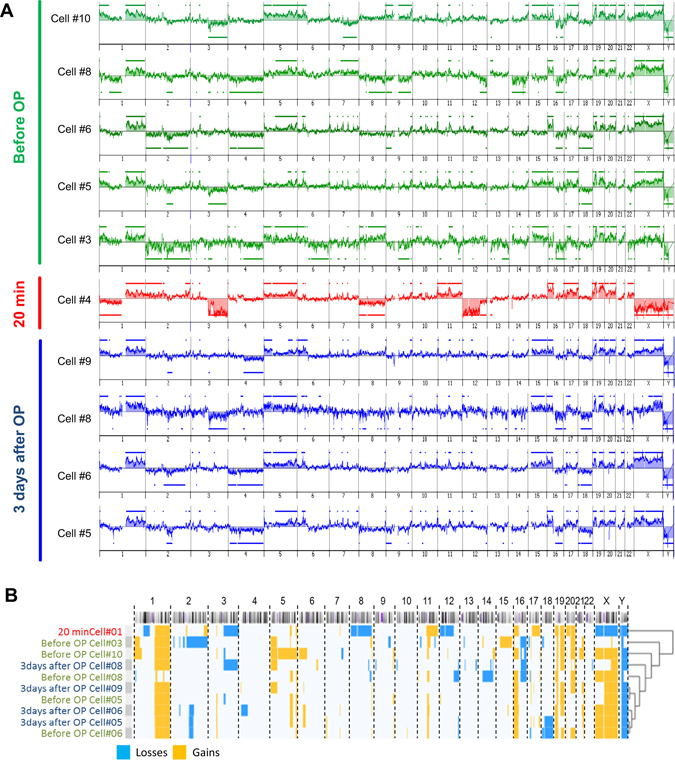
(**A**) Genomic landscape, analysed by array comparative genomic hybridization (aCGH), of individually picked CTCs at the three different time-points before and after cement augmentation. Genomic losses are shown below and gains with bars above. (**B**) Hierarchical clustering of genomic alterations demonstrate a distinct molecular of the mechanically released CTC at 20 minutes after cement augmentation.

In order to determine that the released EpCAM+/K+ cells were indeed tumour cells, as well as to determine whether tumour cells with different genomic profiles are released upon cement augmentation, we performed single cell genomic analysis by array CGH of one representative patient after whole genome amplification. Ten CTCs were analysed from patient #4 (Table 1), five preoperative, one 20 minutes postoperative and four after 3 days. Data analyses show that the CTC found 20 minutes post-OP presents a very different genomic landscape compared to the CTC present pre-operatively and after 3 days. Many of the alteration present in the preOP CTCs were shared with the CTCs taken after 3 days. In contrast, we detected new alterations such as deletion of whole chromosome 8 and partial deletion of 3, 12 and amplification of 17 only in the 20 minutes CTC (Fig. 3A). Clustering analysis of the 10 CTCs highlights the genomic segregation of the 20 minutes CTC from the other CTC due to a completely diverse pattern of genomic abnormality (Fig. 3B).

## Discussion

Mechanical influences have long been suspected to enhance the release of viable tumour cells into the circulation^23–25^. With increasing numbers of long-term cancer survivors, 5–10% of cancer patients will develop spinal metastases during their disease course^26^. However, cancer metastases are still amenable to salvage therapy with a relatively good prognosis compared to earlier decades, especially in an metastatic setting where systemic metastatic spread is limited to one organ system, or even to a solitary metastasis^27^. It is therefore crucial to re-evaluate current surgical treatment options for symptoms in the palliative overall disease context and the recent advances in oncological therapy. In our study, it is demonstrated for the first time that cement augmentation via vertebro- and kyphoplasty for the treatment of spinal column metastasis leads to a temporal, but significant haematogenous release of CTCs.

Cement augmentation due to VP or KP represents safe approaches to treat fractured and/or symptomatic spinal metastasis. Compared to an open surgical approach, serious complications are rarely seen in vertebroplasty and patients vastly benefit from the short procedure^16, 26^. As quality of life can be improved in many cases, overall 81% of patients are satisfied with the treatment outcome^28^. However, cement leakage is a known complication of cement augmentation^16^. The study by Barragán-Campos *et al*. demonstrated that cement leaks predominantly into the vasculature, compared to non-vascular structures (78.5% vs. 21.5%)^29^. In addition, spinal metastasis are very well vascularised^15^. Whether this results in a special vulnerability to serve as an organ of origin for CTC dissemination due to mechanical stress during VP has not been investigate so far. In this study, we detected significantly increased CTC counts after KP/VP. In order to confirm that mechanically disseminated tumour cells were able to pass through capillary beds and enter the circulation, we chose the time-point of 20 minutes after cement augmentation. Interestingly, in a few patients we were able to observe numerous large CTC cluster at this time-point, indicating, that even large tumour cell aggregates are able to circulate. Although, it was initially assumed that CTC clusters are too big to extravasate, investigations, including a study by Au *et al*., demonstrated that these cells indeed can leave the blood vessels and home to distant organs^30^. In our case, CTC clusters confirm the pathophysiology of a mechanical distribution, as large clusters most likely originated due to mechanical forces or cement associated heat-induced disruption of the metastatic tumour environment. Interestingly, in a subgroup analysis of non-breast cancer patients, kyphoplasty showed a higher CTC release compared to vertebroplasty. If this is the result of increased intrametastatic pressure of the kyphoplasty and/or reflects differences in metastatic tissue density and composition will be subject of investigation in upcoming studies.

Formation of distant metastases after surgical treatment of the primary tumour is generally considered to be caused by undetected micrometastatic cells, which have already disseminated to distant organs such as the bone marrow before the surgical resection of the primary tumour^1^. Earlier investigations have already suggested that diagnostic intervention or surgery itself could promote metastasis formation^22^. Studies investigating colorectal cancer patients describe that tumour cell dissemination can be increased due to surgery^25^, colonoscopy and endorectal ultrasound^23, 24^. Analogous findings of increased tumour cell mRNA were made for radiofrequency ablation in malignant lung tumours^31^, or surgery for hepatocellular carcinoma^32^. In pancreatic cancers, the observation that 83% of patients exhibited increased postoperative CTC counts after standard surgery compared to no-touch surgery, further increased awareness for the topic of intraoperative tumour cell seeding^33^. Interestingly, in a mouse model Juratli *et al*. demonstrated that biopsy, but not tumour resection or simple compression of the tumour mass may result in increased CTC counts, pointing towards a critical role of disruption of the tumour mass integrity that can cause a mechanical release^34^.

Cement augmentation via KP or VP increases the intrametastatic pressure and hypothetically forces tumours cells into the surrounding blood vessels. Although a study by Roedel *et al*. demonstrated that local tumour progression in the spine after simple cement augmentation is seen in 14% of breast-cancer patients, which was not influenced by radiotherapy^35^, paraspinal metastases and local tumour recurrences are rare^18^. Yet, in this study 86% of patients in the augmented breast-cancer cohort developed new distant metastases^35^. This is in line with our observation, as 72.2% of patients developed new metastases after the cement augmentation procedure. Unfortunately, due to the clinical heterogeneity we cannot compare the rate of new metastatic seeding with a matching control cohort. Nevertheless, it is important to note, that, although many patients were in an oligometastatic disease setting, i.e. patients who only presented with clinically apparent metastases in one organ system such as the bone, developed additional metastases after the cement augmentation. Oligometastatic patients are the patient group that could suffer the most from an iatrogenic distribution of tumour cells, as metastatic spread at the time of operation presumably has been limited to only one organ system. CTC dissemination after cement augmentation could therefore present a serious disadvantage for patients with limited systemic tumour cell infestation. However, proving this hypothesis is difficult, as the released CTCs would have to be labelled during cement augmentation, or potentially induced metastases would need to be biopsied in order to confirm their clonal origin using whole genome sequencing. Although, our series was able to demonstrate the mechanically induced CTC dissemination, a large clinical registry is needed to provide sufficient power to dissect the diverse disease heterogeneity to unequivocally confirm the clinical impact of CTC release during cement augmentation.

There are currently no longitudinal human studies available, which could unravel to what extent mechanically released tumour cells have the capacity, to form distant metastases. Early studies in colorectal cancer, however, have shown that if a “no-touch” surgery with prior vessel ligation before *en-bloc* tumour removal is performed, can affect the distribution of metastatic seeding, result in decreased metastatic spread to distant organs and can have a profound impact on patient survival^36, 37^. The discovery is currently being evaluated in a large prospectively randomized trial^38^. Animal studies have shown that only a minute proportion of tumour cells are capable of forming metastases and that the great majority of CTC are cleared from the circulation within 24 hours^39^. In the present study we show that the mechanically disseminated CTC is genomically different from the original population. Although, technical limitation due to the cell picking and WGA prevented us to analyse additional CTCs, the clearly distinct molecular profile that we found in CTC at 20 minutes post-OP, indicates that VP/KP could potentially change the CTC population in the blood stream, not only in number but also in genomic subtype, leading to uncertain outcome concerning the metastatic spread. Our data open the discussion whether additional therapeutic options could reduce the seeding of viable tumour cells such as pre-operative radiation^18^, kypho-IORT^40^, or radiofrequency ablation of the spinal metastases before cement augmentation^41^. Another approach that could be considered in the future is the perioperative application of targeted therapeutics, tumour-specific antibodies or cellular components which specifically interfere with the metastatic cascade, i.e. adhesion. Here, for example, TNF-related apoptosis inducing ligand (TRAIL)-coated leukocytes or genetically engineered platelets have shown promising effects to neutralize CTCs *in vitro* and *in vivo* ^42, 43^. An additional option which has been investigated is the transient implant of nanostructured surfaces with immobilized nanotubes or a scaffold to capture and trap CTCs without requiring tumour specific surface markers for identification^44, 45^. Upcoming studies on this subject will also have to discuss the role of peri- and postoperative chemotherapy and identify if CTCs can potentially serve as intraoperative biomarker in this decision making process^46^.

Taken together, this is the first study to report that peripheral CTC are temporarily significantly increased due to the vertebral augmentation procedure. Our findings give new insights into the biological dynamics of CTC dissemination and provide a rationale for the development of new prophylactic strategies to reduce the increased mechanical dissemination of CTC after vertebroplasty.

## Materials and Methods

### Patients and Study design

In this study, we prospectively enrolled 24 patients with metastatic involvement of the spinal column (German Clinical Trial Register: DRKS00007730, 26/01/2015). Three patients were excluded from the analysis after no CTCs could be detected at any given time-point. Informed consent was obtained from all patients. The study was approved by the medical ethics committee of the Chamber of Physicians of Hamburg. All experiments were performed in accordance with local guidelines and regulations. Peripheral blood samples for CTC analyses were obtained in all patients at three time points: preoperatively, 20 minutes post-cement augmentation and 3–5 days post-operatively. In five patients a fourth sample was collected during follow-up visits (>6 months after vertebroplasty).

For the vertebroplasty procedure (*N* = 11), a 13-gauge needle was advanced to the central aspect of the lesion at the vertebral body. Cement (VertaPlex HV, Stryker, Duisburg, Germany) was prepared on the bench and infused under lateral and anterior-posterior (ap) fluoroscopy into the vertebral body. Infusion was stopped when the cement reached to the posterior aspect of the vertebral body or entered an extraosseous space, such as the intervertebral disk or an epidural or paravertebral vein. For kyphoplasty (*N* = 10), balloon dilatation was performed before cement was applied. Two patients received an intraoperative radiotherapy in addition to kyphoplasty (KyphoIORT) as described previously^40^.

### Detection of circulating tumour cells

For CTC quantification, 7.5 ml peripheral whole blood was collected in CellSave tubes (Immunicon, Inc., Huntingdon Valley, PA). The semi-automated analysis was performed as described elsewhere^19^. Blood samples were kept at room temperature for ≤72 hours before analysis using the CellSearch™ assay (CellSearch™ Epithelial Cell Kit/CellSpotter™ Analyser, Menarini-Silicon Biosystems, San Diege, CA, USA). The assay uses a ferrofluid coated with antibodies to epithelial cell adhesion molecule (EpCAM) to immunomagnetically separate cells of epithelial origin from blood, and fluorescent staining to differentiate between debris, hematopoietic cells, and epithelial-derived circulating tumour cells^19^. CTCs quantified and characterised in this study were cells with a positive staining for keratins (K) and nuclear DAPI staining, but negative for the pan-leukocyte marker CD45. The accuracy and reproducibility of the CellSearch system have been described previously^19, 47^.

### Whole genome amplification and array CGH analyses

Single keratin positive CTCs were picked in 200 µl PCR tubes using a micromanipulator as described previously^48^. DNA of the picked CTCs was amplified using the *Ampli*1 whole genome amplification (WGA) kit (Silicon Biosystems, Castel Maggiore, Italy). Array based comparative genomic hybridisation (aCGH) was performed on a SurePrint G3 Human CGH Microarray 4 × 180k Agilent platform as previously described on CTCs obtained from the same patient at three different time points^49, 50^. As reference DNA we used a pool of WGA products obtained from single CD45^pos^ cells isolated from CellSearch^TM^ samples from male healthy donors. The analysis presented here, employed the ADM‐2 algorithm with a threshold of 6.5. For data centralisation we used the diploid peak method and for filtering aberrations we considered regions with a minimum of 250 probes and a minimum absolute mean log_2_ ratio of 0.45.

### Data analysis and statistics

Statistical analyses were performed with Matlab R2016a (The Mathworks). A generalized linear mixed-effect model for repeated measures was used in combination with backward elimination of variables, to correlated the CTC counts with 1) time point of blood collection (before/after 20 minutes/after 3–5 days), 2) procedure (kyphoplasty/vertebroplasty), 3) tumour entity (breast cancer/other), 4) cement volume, 5) metastasis volume 6) systemic metastasis (oligo/multi), and 7) metastasis location (thoracic/lumbar), and interaction effects. The model’s distribution was Poisson and was corrected for age. Survival analysis was carried out using Kaplan-Meier curves and log-rank test. Correlative analysis was performed using Spearman correlation. For calling statistical significance, alpha of 0.05 was applied in all analyses. *P*-value < 0.05 was considered as statistically significant. All graphical and statistical analyses were performed using the GraphPad Prism™ 5.0 and SPSS™ version 18.0 software (SPSS, Chicago, IL, USA). All data generated or analysed during this study are included in this published article (and its Supplementary Information files).

## Electronic supplementary material

Supplemental Information
