# Supplementary material for: Circulating Tumour Cell Release after Cement Augmentation of Vertebral Metastases
Source: Sci Rep. 2017 Aug 3;7:7196. doi: 10.1038/s41598-017-07649-z (PMC5543076; doi:10.1038/s41598-017-07649-z)
Supplement: Supplementary file 1 — Supplemental Information [file 41598_2017_7649_MOESM1_ESM.pdf]

Circulating Tumour Cell Release after Cement Augmentation of Vertebral Metastases

Malte Mohme[1*], Sabine Riethdorf[2], Marc Dreimann[3], Stefan Werner[2], Cecile L. Maire[1], Simon A. Joosse[2], Frederic Bludau[4], Volkmar Mueller[5], Rui P. L. Neves[6], Nikolas H. Stoecklein[6], Katrin Lamszus[1], Manfred Westphal[1], Klaus Pantel[2], Harriet Wikman[2‡], Sven O. Eicker[1‡]

[1] Department of Neurosurgery, University Medical Centre, Hamburg-Eppendorf, Germany
[2] Department of Tumour Biology, University Medical Centre Hamburg-Eppendorf, Germany
[3] Department of Trauma-, Hand- and Reconstructive Surgery, University Medical Centre Hamburg-Eppendorf, Germany
[4] Department for Trauma Surgery, University Medical Centre Mannheim, University of Heidelberg, Germany
[5] Department of Gynecology, University Medical Centre Hamburg-Eppendorf, Hamburg, Germany
[6] Department of General, Visceral and Pediatric Surgery, University Hospital and Medical Faculty of the Heinrich-Heine University Dusseldorf, Dusseldorf, Germany

‡ these authors contributed equally
* corresponding author

Corresponding Author:
Malte Mohme, M.D.
Department of Neurosurgery
University Hospital Hamburg-Eppendorf
Martinistr. 52, 20246 Hamburg, Germany
Tel.: +49 (0) 40 7410-0
Fax: +49 (0) 40 7410-58121
m.mohme@uke.de

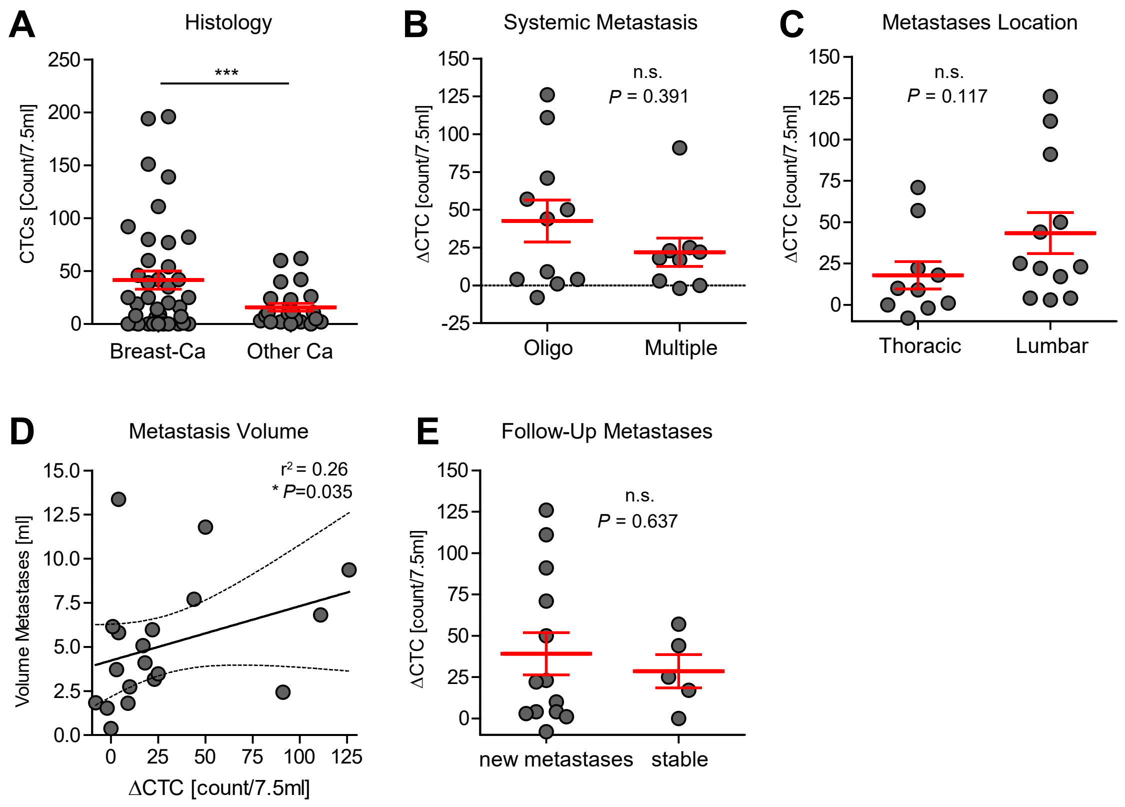
A
Histology
***
CTCs [Count/7.5ml]
250
200
150
100
50
0
Breast-Ca
Other Ca
B
Systemic Metastasis
n.s.
P = 0.391
ΔCTC [count/7.5ml]
150
125
100
75
50
25
0
-25
Oligo
Multiple
C
Metastases Location
n.s.
P = 0.117
ΔCTC [count/7.5ml]
150
125
100
75
50
25
0
Thoracic
Lumbar
D
Metastasis Volume
r² = 0.26
* P=0.035
Volume Metastases [ml]
15.0
12.5
10.0
7.5
5.0
2.5
0.0
0
25
50
75
100
125
ΔCTC [count/7.5ml]
E
Follow-Up Metastases
n.s.
P = 0.637
ΔCTC [count/7.5ml]
150
125
100
75
50
25
0
new metastases
stable

Supplementary Figure 1

A) CTC counts (CTC/7.5ml blood) of all three measurements (preOP, 20min, 3-5 days postOP) stratified by Histology. B) Increase of CTC counts (CTC_{preOP} – CTC_{20min} = ΔCTC/7.5ml) compared between patients with only one organ system affected by metastasis (Oligo) versus patients with metastasis in multiple organ systems (Multiple). C) Comparison of CTC release (ΔCTC) by location of metastasis within the spinal column. Bars and error depict mean and standard error (SE). D) Correlative comparison of CTC increase (ΔCTC) with metastasis volume [ml] with linear regression curve fit and 95% CI analysed by Spearman correlation. E) Comparison of CTC release (ΔCTC) by development of new metastasis during follow-up.

Supplemental Table 1. Breast-Ca Patient Characteristics

No.	Primary Tumor	Follow-up from VP [days]	death	Pathology Primary Tumor							Pathology Vertebral Metastasis				Metastatic spread*	Location of other Metastases
				ER	PR	Her2	T	N	M	G	ER	PR	Her2	GATA3		
1	Breast-Ca	812	yes	+	+	-	pT1c	pN1	M0	2	+	-	-	+	oligo	bone
2	Breast-Ca	710	no	+	+	-	pT2m	pN2a	M0	3	+	-	-	+	oligo	bone
3	Breast-Ca	338	yes	-	-	n.a.	pT1c	pN1	M0	n.a.	+	-	-	n.a.	multiple	liver, lymph nodes
4	Breast-Ca	n.a.	n.a.	+	+	-	pT1c	pN1	M0	2	n.a.	n.a.	n.a.	n.a.	multiple	bone, stomach
5	Breast-Ca	446	no	-	-	n.a.	pT2	pN0	M0	2	n.a.	n.a.	n.a.	n.a.	multiple	bone, lung
6	Breast-Ca	n.a.	n.a.	n.a.	n.a.	n.a.	n.a.	n.a.	n.a.	n.a.	+	-	-	+	oligo	bone
7	Breast-Ca	412	no	+	+	n.a.	pT1c	pN1	M0	3	n.a.	n.a.	n.a.	n.a.	oligo	bone
8	Breast-Ca	250	no	+	+	-	pT2	pN0	M0	2	+	+	-	+	oligo	bone
9	Breast-Ca	178	no	+	+	-	pT3	pN2a	M0	2	n.a.	n.a.	n.a.	n.a.	oligo	bone
10	Breast-Ca	301	no	-	-	+	cT1	cN0	cM0	2	n.a.	n.a.	-	+	oligo	bone
11	Breast-Ca	303	no	+	+	-	pT1c	pN2a	M0	2	n.a.	n.a.	n.a.	+	multiple	bone
12	Breast-Ca	174	no	n.a.	n.a.	n.a.	n.a.	n.a.	n.a.	n.a.	-	-	-	+	oligo	bone
13	Breast-Ca	147	no	+	+	-	pT3	pN1a	pM1	2	n.a.	n.a.	n.a.	n.a.	multiple	bone, liver, lung, lymph node

* Oligo = only one organ system affected

Supplemental Table 1: Characteristics of breast-cancer patients. ER: estrogen receptor, PR: progesterone receptor, TNM stages, n.a.: not available, oligo: only one organ system affected

Supplemental Table 2. Other Tumor Entities – Patient Characteristics

No.	Primary Tumor	Follow-up from VP [days]	death	Pathology Primary Tumor					Pathology Vertebral Metastasis				Metastatic spread*	Location of other Metastases
				Histology	T	N	M	G	AE1/AE3	CK7	CK5/6	TTF-1		
14	NSCLC	239	no	Adenocarcinoma	cT4	cN2	cM1	n.a.	+	+	-	+	multiple	bone, lung, liver
15	NSCLC	13	yes	Adenocarcinoma	n.a.	n.a.	n.a.	n.a.	+	+	-	+	oligo	bone
16	NSCLC	449	no	Adenocarcinoma	n.a.	n.a.	n.a.	n.a.	+	+	-	+	oligo	bone
17	NSCLC	374	no	Adenocarcinoma	cT2	cN2	pM1	n.a.	n.a	n.a	n.a	+	oligo	bone
18	NSCLC	118	no	Adenocarcinoma	cT1a	cN0	pM1b	n.a.	+	+	-	-	oligo	bone
19	Colorectal-Ca	80	n.a.	-	pT4a	pN2a	M1	3	n.a	n.a	n.a	n.a	multiple	liver, lung, lymph node
20	Urothel-Ca	132	n.a.	-	cT4	cN2	cM1	n.a.	n.a	n.a.	+	n.a	multiple	bone, lung, lymph node
21	Prostate-Ca	577	no	-	n.a.	n.a.	n.a.	n.a.	+	-	n.a	-	multiple	bone

* Oligo = only one organ system affected

Supplemental Table 2: Characteristics of non-breast-cancer patients. NSCLC: non-small cell lung-cancer, TNM stages, n.a.: not available, oligo: only one organ system affected
